# Dentoalveolar class III treatment using retromolar miniscrew anchorage

**DOI:** 10.1186/2196-1042-14-7

**Published:** 2013-05-23

**Authors:** Laura Poletti, Aimara A Silvera, Luis T Huanca Ghislanzoni

**Affiliations:** Department of Orthodontics, IRCCS Cà Granda - Ospedale Maggiore Policlinico, University of Milan, Milan, Italy; Private practice, Ponte San Pietro, Italy; Private practice, Aosta, Italy; Dipartimento di Scienze Biomediche per la Salute, Università degli Studi di Milano, Milano, Italy

## Abstract

In this article, we report the successful use of miniscrews in the distalization of the lower dentition to correct an Angle class III malocclusion with lower anterior crowding in a dolichofacial adult patient. Conventional intraoral and extraoral appliances have many disadvantages, including the need for patient cooperation, potential for anchorage loss, and vertical extrusion of upper molars and lower incisors. Extrusion should be prevented or minimized when treating long-faced patients with reduced overbite. After third molar extractions, miniscrews were placed in the retromolar area. A sliding jig was applied to distalize the lower molars, while the anterior teeth were bonded and retracted secondarily to avoid round tripping. After 18 months of treatment, molar and canine class I relationship with normal overjet and overbite were achieved. In addition, there was an esthetic improvement in the profile with only a small increase of the lower anterior facial height. These results remained stable at a 12-month follow-up.

## Background

Class III malocclusion has a relatively low incidence which varies with race: 1% to 5% among Caucasians [1–3] (5% among Italian people) [4], 14% in Asians, and 5% to 8% among black people [5]. An accurate diagnosis must distinguish between skeletal and dentoalveolar discrepancies (or a combination of both) to establish an adequate treatment plan. When considering adult patients, if the discrepancy is partly or completely skeletal, orthognathic surgery may be the only way to achieve both an ideal occlusion and an esthetic outcome. If the discrepancy is mainly dentoalveolar, orthodontics alone can achieve a good and stable occlusion, but not significant improvements in the profile, which are prerogatives of orthognathic treatment [6]. Surgery, however, entails biological and economical costs that require a strong motivation of the patient.

Distalization of the mandibular dentition is a viable way to correct a class III anteroposterior relationship (a negative overjet or an edge-to-edge occlusion). It can be achieved in two different manners: extractions in the anterior or middle arch (two premolars or one central incisor depending on crowding and entity of overjet) [7, 8] or molar extractions (third molars or less frequently second molars) and whole arch distalization [9–13]. Mandibular molar distalization is a highly difficult movement to obtain in orthodontics [14]. Different devices have been used to reach this goal, including mandibular headgear, lip bumper, distal extension lingual arch, Jones jig, Franzulum appliance, multi-brackets with class III elastics, and multi-loop edgewise therapy [15]. Although these techniques can provide an acceptable incisor relationship and occlusion, they mostly produce a distal tipping with rotation of the molars rather than a bodily distal movement. Furthermore, the quality of treatment results depends on patient cooperation. When considering intraoral devices, the third Newtonian law plays an important role: forces always occur in pairs. The distalization of the molars tends to move the anterior teeth forward, which then have to be retracted against the distalized molars. These mechanics expose the anterior teeth to dangerous jiggling forces [16, 17]. Moreover, the teeth are subjected to forces in all three planes of space via class III elastics causing changes in the transverse dimension, in addition to extrusion of the lower incisors and upper molars, resulting in an increased lower anterior facial height [12, 18]. Such effect should be avoided in long-face patients with reduced overbite.

Dental implants [19], miniscrews [20], and miniplates [15] can provide absolute anchorage to prevent these undesirable side effects. In particular, miniscrews are the first choice when looking for skeletal anchorage because of the easy surgical placement and removal procedure, low cost, and multiple placement sites. These temporary anchorage devices (TADs) are useful for various tooth movements, including intrusion, retraction, and protraction. Recently, implant anchorage has been used successfully in a wide variety of adult malocclusions, including class III and open bites [21–23]. This case report describes the distalization of the mandibular dentition with miniscrews in the retromolar area to treat a dental class III malocclusion with anterior crowding and a reduced overbite of 0.5 mm in a long-face adult patient.

## Case presentation

### Diagnosis

**Figure 1:**
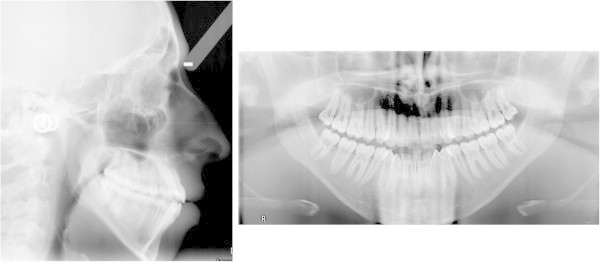
**Pretreatment cephalogram and panoramic radiograph.**

**Figure 2:**
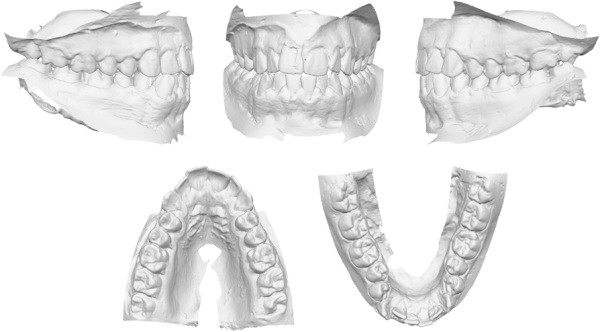
**Pretreatment digital dental casts.**

A young patient, aged 17 years and 7 months, came to the private practice of one of the authors with the chief complaint of dental crowding and esthetic improvement. A dolichofacial appearance with a convex profile was observed (Figure 1). Mild crowding was present, especially in the anterior of the mandibular arch, with a labially positioned right canine. Both arches were narrow and tapered. The molar and canine relationship was class III bilaterally (Figure 2). Periodontal conditions were good with acceptable oral hygiene. The cephalometric tracing showed a skeletal class I (Wits −1.0 mm) but dentoalveolar class III. A hyperdivergent facial pattern (Frankfurt to mandibular plane 37.3°) was present (Table 1).

**Table 1:** **Cephalometric measurements**

	Pretreatment	Posttreatment
Sagittal skeletal		
ANB (°)	5.2	5.6
SNA (°)	81.0	86.0
SNB (°)	75.8	74.9
Wits (mm)	−1.0	−1.4
Co-A (mm)	85.6	86.3
Co-Gn (mm)	115.6	116.9
Vertical skeletal		
FH-NL (°)	2.5	5.1
FH-OP (°)	8.4	11.9
NL-ML (°)	34.7	35.9
FH-ML (°)	37.3	41.1
N-SNA (mm)	59.1	61.1
SNA-Me (mm)	70.5	71.5
Co-Go-Me (°)	143.4	145.5
Interdental		
Overjet (mm)	0.8	1.6
Overbite (mm)	0.3	1.8
Molar relationship (mm)	7.4	2.6
Interincisal angle (°)	127.6	129.0
Maxillary dentoalveolar		
U1 to FH (°)	116.0	113.5
U1 hortizontal (mm)	47.8	48.3
U1 vertical (mm)	25.2	25.5
U6 horizontal (mm)	17.9	20.5
U6 vertical (mm)	21.4	21.5
Mandibular dentoalveolar		
IMPA (°)	79.1	76.4
L1 horizontal (mm)	66.6	64.5
L1 vertical (mm)	37.5	39.1
L6 horizontal (mm)	44.8	40.8
L6 vertical (mm)	25.9	25.7

### Treatment objectives

The objectives of treatment were to obtain a class I molar and canine relationship with ideal overjet and overbite, relieve the crowding in the mandibular arch, and maintain the lower anterior facial height. Therefore, we planned to extract all of the third molars and use titanium miniscrews placed in the retromolar area for anchorage to move the mandibular dentition distally. Special care was dedicated to avoid second molar extrusion during the distal movement to prevent the undesirable wedge effect, which potentially increases facial height.

### Treatment progress

**Figure 3:**
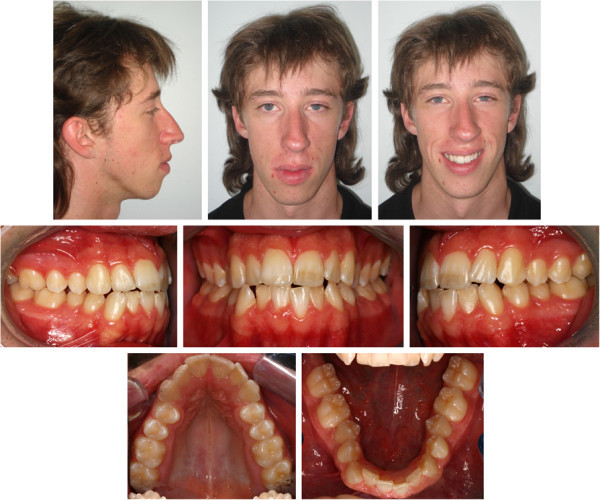
**Pretreatment photographs after third molar extractions.**

**Figure 4:**
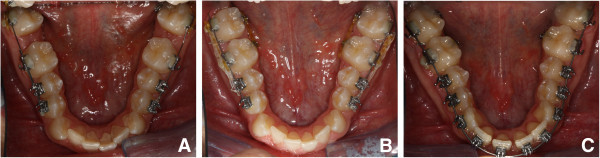
**Occlusal intraoral photographs during treatment showing progressive distalization.** (**A**) At 2 months, (**B**) at 6 months, and (**C**) at 8 months.

The upper and lower third molars were extracted 6 months before the start of the active treatment to allow for bone healing in the extraction area (Figure 3). Two 1.5-mm diameter miniscrews were then placed into the retromolar area (11 mm on the left and 14 mm on the right due to different soft tissue thickness). They were immediately loaded with light forces directed to the second molars. A segmented arch approach was used including both the lower molars and premolars (Figure 4A). The canines and incisors were excluded in the first distalizing phase of the treatment to avoid round tripping (proclination of the crowded incisors followed by retraction later in treatment).

**Figure 5:**
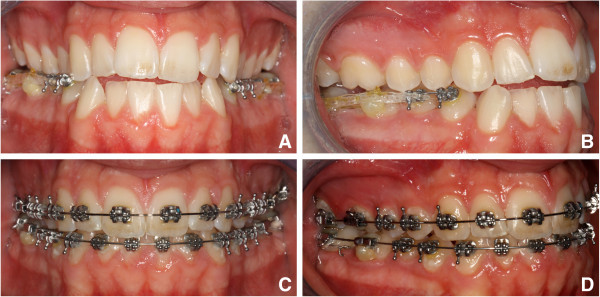
**Fronto-lateral intraoral photographs during treatment showing open bite development and self-correction.** (**A**, **B**) At 6 months and (**C**, **D**) at 14 months.

After achieving the desired distal movement of the second molars, they were tied with steel ligatures to the miniscrew head and used as indirect anchorage to distalize the rest of the arch. The elastic traction was moved to the first molars and then to the first premolars (Figure 4B). During this phase, an open bite developed (from 0.3 to −2.0 mm of overbite) due to a small amount of extrusion and unbalanced occlusal contacts at the premolar level (Figure 5A). At this time, with the lower incisors naturally unraveled as a consequence of sagittal space gain, both the lower anteriors and the upper arch were bonded (Figure 4C). Minor extrusion of the lower incisors and an improved balance of occlusal contacts in the molar and premolar area accounted for an overbite restoration of 1.8 mm, without the use of vertical elastics (Figure 5B).

**Figure 6:**
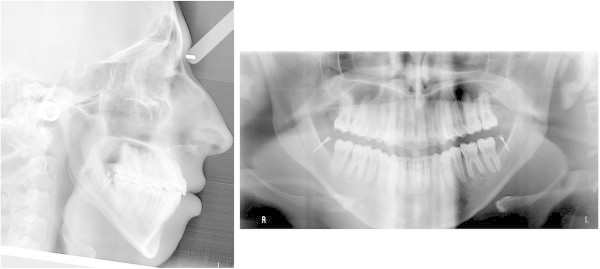
**Posttreatment cephalogram and panoramic radiograph with miniscrew inserted.**

**Figure 7:**
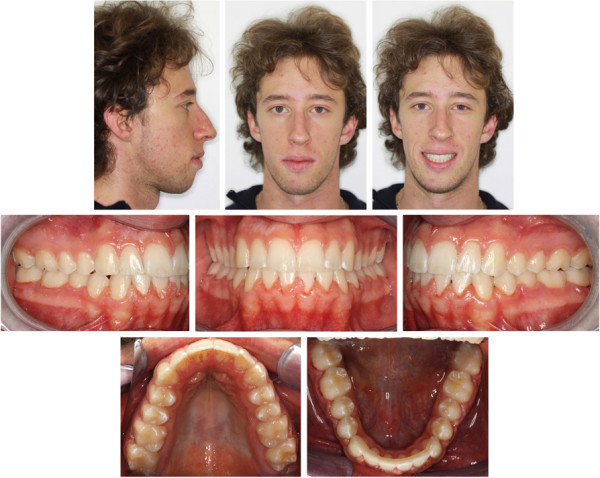
**Posttreatment photographs.**

**Figure 8:**
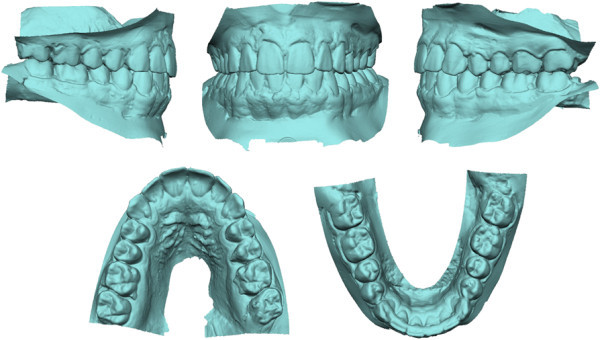
**Posttreatment digital dental casts.**

Interarch elastics were avoided to prevent extrusion in an open bite patient. The treatment lasted 18 months. A lower splint was bonded in the mandibular arch, and an upper removable plate was delivered as retention for the maxillary arch (Figures 6, 7, 8).

## Discussion

When deciding to treat an open bite case without surgery, the main goal should be to avoid further opening of the bite. Any sagittal movement of the teeth will account for at least a mild amount of extrusion; therefore, it is best to avoid interarch elastics which have a vertical component of force. This was the rationale for the intra-arch mechanics used in this case to distalize the lower dentition with TADs as anchorage.

Miniplates and miniscrews have similar success rates, and both provide enough anchorage to distalize the mandibular molars [21]. We preferred, however, to use miniscrews because of the easier placement and removal technique which does not require flap surgery, resulting in less discomfort for the patient and a faster healing period. We chose to place TADs in the retromolar area because this is a site with a relatively thick cortical bone layer, far from dental roots, and does not interfere with dental movements [23, 24]. TADs were placed 6 months after third molar extractions as placing miniscrews too early could lead to miniscrew loosening because of inadequate quality of the healing bone to achieve primary stability.

When placing the miniscrews in the retromolar area, the clinician is faced with an area where the soft tissue may be between 3.0 and 6.0 mm thick. Healing after third molar extractions also accounts for an increased thickness of the retromolar mucosa. The orthodontist would like to have the miniscrew as apical as possible with respect to the second molars to achieve vertical control, but this is not possible due to soft tissue limitations. It is not unlikely to notice mucosal overgrowth around the head of the implant; therefore, care must be taken to prevent this from occurring [17]. Because this occurred in our case, once the second molars were properly distalized, they were tied to the miniscrew with a steel ligature and used as indirect anchorage to distalize the rest of the arch.

**Figure 9:**
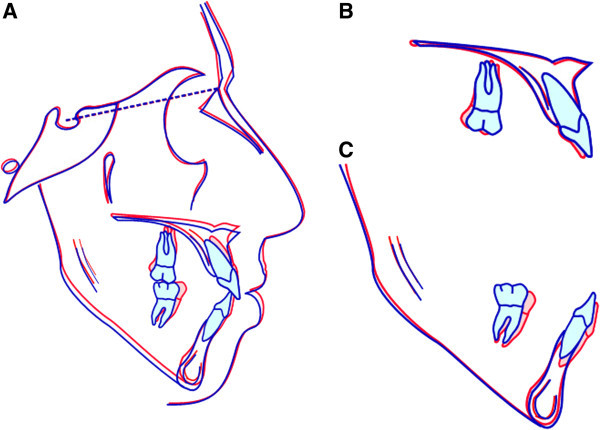
**Superimposed pretreatment (red line) and posttreatment (blue line) cephalometric tracings.** (**A**) On the sella-nasion plane at sella, (**B**) on the palatal stable structures, and (**C**) on the mandibular stable structures.

**Figure 10:**
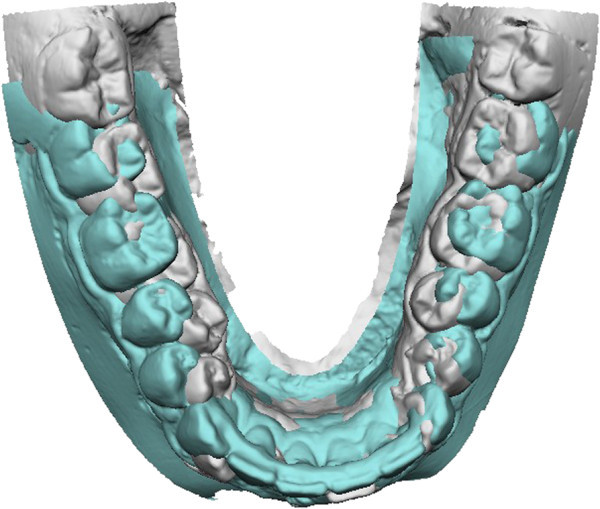
**Superimposed pretreatment (white) and posttreatment (green) three-dimensional dental casts with occlusal view showing distalization.**

Two fundamental methods of applying distalizing forces are reported in the literature: a tooth-by-tooth distalization or an *en masse* distalization. The latter may be performed by applying a direct reactive force to the first premolars, canines, or to anterior hooks [15, 16]. In our case, the molars were distalized one by one, followed by the bicuspids to allow a fast distal movement of each tooth. The sectional wire acted as a sliding guide which prevented rotations and excessive tipping [16]. In our patient, the lower first molars were distalized 4.0 mm relatively to the mandibular plane, with a distal tipping of approximately 10° (Figures 9 and 10). The molar distalization occurred as a rotation around the apex of the distal root, as is evident from mandibular superimpositions. A segmented arch approach gave us some control in the vertical dimension in the molar area, while the premolars exhibited some extrusion, as shown by ‘root shadows’ in the final panorax (Figure 6). An open bite developed in the first part of the treatment: the overbite went from 0.3 to −2.0 mm and then to 1.8 mm (Figure 5), without any interarch mechanics. This can be seen as a consequence of the initial drifting of the contacts in the premolar area (due to extrusion) and a subsequent settling of the occlusal contacts once the lower arch assumed its final shape (decompensation through vestibular of a tapered arch and improved occlusal contacts).

The anterior teeth were bonded secondarily to avoid round tripping, i.e., the incisors tipping facially followed by retraction created by the distalization of the buccal teeth. Bicuspid extractions were avoided as intercuspation of the bicuspids is recognized as a stability factor [13, 25].

The face exhibited a slight tendency towards elongation. Intermaxillary divergence increased by only 1.2° as a direct consequence of orthodontic biomechanics, while the Frankfurt to nasal plane increased by 2.6° (showing a tendency towards unfavorable growth), and the Frankfurt to mandibular plane increased by 3.8° (from 37.3° to 41.1°). The unfavorable skeletal pattern was properly counteracted at the occlusal level where the molars exhibited 4.8 mm of correction in molar relationship from a class III relationship to a sound class I relationship. In relation to their bone bases, both the upper and lower molar did not extrude, while there was some extrusion of the lower incisors (1.6 mm). As suggested by Yanagita et al. [22], the symphysis was remodeled, and both the skeletal and soft tissue B points were deepened, resulting in a more relaxed lower lip.

Until now, little information has been available about the posttreatment stability of orthodontic treatment using implant anchorage [10]. Sugawara et al. [15] found minimal short-term relapse, and no significant correlation was found between the amount of relapse and tipping ratio and the amount of tooth movement. Lima and Lima [11] showed 4 years of stable retention after distalization of the mandibular dentition in the treatment of class III open bite adult patients. Many factors may affect posttreatment stability, including prolonged or permanent retention, maintaining pretreatment arch form and intercanine width, obtaining proper occlusal relationship and function, and taking into account muscle balance and harmony [26, 27]. In our case, we managed retention with a lower bonded splint and an upper removable plate. Miniscrew removal was deferred to permit an immediate correction of any eventual relapse. At 1 year posttreatment, no relapse had occurred, and the TADs were removed.

## Conclusions

Mandibular arch distalization through TADs inserted in the retromolar area after extraction of the third molars appears to be a viable option when treating a dentoalveolar class III patient with lower anterior crowding.

## Consent

Written informed consent was obtained from the patient for publication of this report and any accompanying images.
